# Mapping Consumer Preference for Vegan and Omnivorous Diets for the Sensory Attributes of Flour Products with Iodine-Fortified Plant-Based Ingredients

**DOI:** 10.3390/nu16244392

**Published:** 2024-12-20

**Authors:** Krystyna Szymandera-Buszka, Agata Jankowska, Anna Jędrusek-Golińska

**Affiliations:** Department of Gastronomy Science and Functional Foods, Faculty of Food Science and Nutrition, Poznań University of Life Sciences, Wojska Polskiego 31, 60-624 Poznań, Poland; agata.jankowska@up.poznan.pl (A.J.); anna.jedrusek-golinska@up.poznan.pl (A.J.-G.)

**Keywords:** sensory analysis, consumer analysis, food fortification, flour products, vegetables, vegan diet, omnivore diet

## Abstract

Objectives: Flour products with iodine-fortified dried vegetables can be a good source of iodine. However, in addition to iodine stability, the sensory quality of these products is also important. Therefore, this study aimed to assess the effect of adding iodine-fortified dried vegetables to flour products (gnocchi and ciabatta) on their sensory quality and map consumers (vegan/omnivore diets) as potential consumers of fortified flour products with iodine-fortified dried vegetables. Methods: A quantitative analysis of the sensory desirability and profiling were used to achieve the work objective. Results: It was confirmed that there was no relationship between the form of iodine (without iodine/KIO_3_/KI) and the intensity of all definite descriptors of colour, aroma, and taste. We also confirmed the relationship between the vegetable type and the descriptors’ intensity. It was confirmed that the positive attribute with the highest impact was the pumpkin taste, and the attribute with the most negative impact was the cauliflower aroma. This was true for consumers on vegan and omnivore diets, and they confirmed the most positive attitude toward the taste of pumpkin. Conclusions: Introducing dried iodine-fortified vegetables (gnocchi dumplings 24%; ciabatta rolls 5%) allows for the high sensory desirability of designed products. Introduced iodine (gnocchi dumplings 30 mg I/100 g; ciabatta rolls 9–11 mg I/100 g) does not change products’ sensory profiles.

## 1. Introduction

Iodine deficiency is a common public health problem in many countries worldwide, both in developing countries and Western countries with highly developed health services [1]. A deficiency of this element is dangerous for all age groups, especially for pregnant women, children, and people on plant-based diets [2,3]. These groups are particularly vulnerable to Iodine Deficiency Disorders (IDDs) due to the increased demand for iodine, in the case of children and pregnant women, and due to the reduced intake of iodine dietary sources and the increased consumption of products providing goitrogenic compounds, in the case of people on plant-based diets [4,5]. Some of the more serious effects of iodine deficiency are hypothyroidism, decreased mental functions, mental and physical underdevelopment of children, miscarriages, and stillbirths [6,7,8].

Therefore, many countries whose societies are at risk of a deficiency of this element have introduced prophylaxis programs [1]. The most frequently used method of preventing iodine deficiency is fortifying food with iodine. Bread, milk, water, and salt are fortified. The most common method is the iodization of table salt, which is recommended by the World Health Organization (WHO) [9,10]. The WHO recommends iodizing salt because it meets all the assumptions of effective prevention, i.e., it is one of the few products consumed by all people, its consumption does not change seasonally, and its fortification is simple to implement, cheap, and easy to control at every stage [11]. This method has brought satisfactory results for many years and allowed many populations to be classified as having a sufficient iodine supply [12]. Although iodizing table salt seems to be an ideal method of preventing an iodine deficiency, salt consumption, especially in excessive amounts, harms health [13].

This prompts research into the possibility of using new products as carriers for iodine [14]. The earlier studies confirmed that vegetables are an alternative to table salt as a matrix for iodine [15]. This element should be consumed daily in the recommended amounts to ensure adequate iodine saturation in the body [16]. An attractive solution may be to enrich flour products with dried vegetables fortified with iodine [3]. This makes the process more ecological and fits into current efforts to reduce waste [17].

These products can be consumed daily by every group of consumers, including people particularly vulnerable to iodine deficiency, e.g., vegans. The consumers of these products may be people on a vegan diet, because they meet the assumptions of plant diets, in which interest is constantly growing and which should be taken into account when planning iodine prevention [18,19]. Many studies have confirmed the health benefits of a balanced vegan diet and a reduced risk of developing certain lifestyle diseases, such as cardiovascular disease, type 2 diabetes, obesity, and non-alcoholic fatty liver disease [20,21]. Compared to other dietary patterns considered health-promoting (Mediterranean diet, DASH), eliminating animal products and increasing the consumption of vegetables and fruits, legumes, nuts, and whole grains were associated with a lower risk of all-cause mortality [22]. Vegans are a group that is more likely to exhibit healthy non-diet habits such as attention to sleep hygiene, higher levels of physical activity, and avoidance of smoking, which also decrease the risk of mortality compared to people following a Mediterranean diet [23,24,25]. Despite its proven significant health benefits, a vegan diet carries the risk of deficiencies in nutrients such as protein, vitamin B12, vitamin D, iron, zinc, selenium, and iodine [20,26]. Previous studies indicate the high stability of iodine from adding dried vegetables fortified with iodine [21]. Moreover, consuming flour products enriched with dried vegetables will increase fibre intake. In addition to iodine stability in food products enriched with iodine, the sensory analysis of these products is also important. The frequency of food consumption is determined by their sensory acceptability and desirability. The issue is whether products fortified with iodine still show good sensory quality and whether consumers accept them. Consumers show lower desirability of a product when it is sensorily unattractive. In this case, other product features, such as low price or brand loyalty, seem to be less important [27,28,29,30,31]. Therefore, in designing new products, the sensory quality of these products is checked in detail to know which product attributes are worth strengthening or masking. Moreover, it is vital to combine the results of consumer assessments (subjective feelings) with the results of a quantitative descriptive analysis (objective methods) [32]. Studies have confirmed that dried vegetables constitute a stable matrix for iodine salts [15,33]. However, to implement the product as an element of iodine prophylaxis, the sensory desirability among the target consumers, particularly those exposed to iodine deficiency, should be examined.

Various sensory methods have been used to obtain information on the sensory characterisation of products and which sensory attributes and the intensity of these attributes could drive consumers’ liking or disliking [34]. So far, these approaches have never been applied to define attributes of flour products with iodine-fortified dried vegetables. This study assessed the possibility of designing sensorily attractive products for vegans and omnivores. This approach allowed us to determine whether the sensory behaviours of these two groups are similar or different.

Considering the abovementioned factors, this study aimed to apply consumer tests and the sensory profiling method to assess the impact of adding iodine-fortified dried vegetables to vegan flour products (gnocchi and ciabatta) on their sensory quality. This analysis also aimed to map consumers on vegan and omnivore diets as potential consumers of fortified flour products with iodine-fortified dried vegetables.

## 2. Materials and Methods

### 2.1. Study Design

Quantitative analysis of sensory desirability and sensory profiling were used to evaluate flour products with iodine-fortified dried vegetables and map consumers on vegan and omnivore diets as potential consumers of these products. The research was conducted in Poznań University of Life Sciences (Poland) from April 2023 to November 2024. The recruitment was completed from January 2023 to December 2023. Recruitment information for vegans and nutrition was distributed on Polish internet forums. Formal dependence was not used to recruit subjects for the study. Before each meeting, the researchers contacted the participants, reminding them of the appointment. The products were prepared in the Gastronomic Laboratory of Poznań University of Life Sciences.

Profiling research was performed with the sensory experts (trained sensory team in sensory sensitivity), and consumer analysis was performed with untrained sensory team consumers. All participants (consumers and experts) evaluated all products.

### 2.2. Material

KI and KIO_3_ constituted the sources of iodine (Merc, Darmstadt, Germany). As a source of vegetables, we used Muscat pumpkin (*Cucurbita maxima* Duch.), cauliflower (*Brassica oleracea* var. *botrytis* L.), carrot (*Daucus carota* L.), broccoli (*Brassica oleracea* L.), and beetroot (*Beta vulgaris* L.).

#### 2.2.1. Carriers Preparation

The vegetables (carrot and pumpkin) were washed, peeled, cut, and steamed (100 °C; 10 min) in a convection oven (Rational, Landsberg am Lech, Germany). Then, the vegetable samples were homogenised (homogeniser—Foss, Hilleroed, Denmark) and underwent impregnation according to the procedure presented in the publication by Zaremba et al. [15]. The impregnation conditions included a hydration ratio of 1:1 (*m*/*v*) and incubation at −76 °C/12 h. Detailed impregnation conditions were described by Jankowska et al. [33].

#### 2.2.2. Products Formulations

Gnocchi dumplings were prepared using potatoes (56%—cooked and grated), wheat flour (type 505–10%), and water (10%) purchased in the retail trade.

To prepare the ciabatta roll dough, wheat flour (type 505–52%), water (41.8%), instant yeast (0.9%), and salt (0.3%) were purchased in the retail trade.

The iodine-fortified dried vegetables were hydrated (at 1:3 ratios) and added at the specified levels for gnocchi dumplings (24%) and ciabatta rolls (5%).

To compare the effect of the iodine-fortified vegetables addition, reference samples were adopted: with the addition of iodized salt (KI/KIO_3_) and without dried vegetables, and with the addition of non-iodized salt and iodine-fortified dried vegetables (hydrated at 1:3 ratios).

The iodine analysis indicated that baked rolls contained iodine at 9–11 ug/100 g of the product and gnocchi dumplings at 30 ug/100 g of the product.

The details of sample preparation were described by Jankowska et al. [35].

### 2.3. Methods

#### 2.3.1. Sensory Analysis

##### Experimental Conditions

Studies were performed following following the Code of Ethics of the World Medical Association (with consent of the Rector’s Committee for the Ethics of Scientific Research Involving Humans No. 3/2023 of 25 May 2023). The research was conducted in an appropriately designed and equipped sensory analysis laboratory [36] at the Department of Gastronomic Technology and Functional Food of the University of Poznań University of Life Sciences. The analysis was conducted in 2023–2024 between 9 a.m. and 3 p.m. The samples were assessed in two sessions—the ciabatta rolls on one day and the dumplings on another day—in the following order of serving: six samples, a break of 0.5 h, six samples, a break of 0.5 h, and another six samples. The samples of the products were evaluated on the same day they were made, and were served on odourless white plates.

The samples were coded with three-digit numbers, and the serving order was random (ANALSENS, v.4; Gdańsk, Poland). Samples with a 30–10 g mass were placed in coded containers (50 mL) with covered lids. Unsweetened black tea (temp. ~40 °C) was used as a taste neutraliser between the samples.

##### Participants

In order to study the mapping of preferences for gnocchi dumplings and ciabatta rolls with dried vegetables enriched with iodine, consumer research and an assessment of the sensory profiling of these products were carried out. The selection of groups was carried out using a stratified method, taking into account the selection of a sample of Polish society in terms of diet type, gender, and age. The exclusion criteria were age (under 18 and over 50), pregnancy, and lactation. The study group consisted of 296 people of both sexes (i.e., 45% men and 55% women) aged 18–60 from various areas of Poland, consuming flour products (with a frequency of at least once a day). The respondents followed a vegan diet (44%) or an omnivorous diet (56%).

##### Methods of Sensory Analysis

A sensory desirability test was used in the consumer evaluation.

Consumers evaluated the products’ colour, aroma, taste, and overall desirability. A continuous and unstructured linear scale of 10 cm with margin denotations of “undesirable”–“highly desirable” was used for the analysis.

Simultaneously, sensory profiling was conducted using a sample’s quantitative descriptive profile description of attributes (colour, aroma, and taste) and their intensity values [37] by an 8-member trained panel (experts). A total of 7 descriptors was adapted for colour, 8 for aroma, and 10 for taste. The intensity of each score was determined using the continuous and unstructured linear scale of 10 cm linear scale with appropriate margin descriptions. Uniform margin denotations were applied for descriptors: “undetectable–very intensive”.

The presented research included consumer research (each of 296 consumers assessed each tested sample once) and sensory research, in which a team of 8 experts assessed the intensity of all descriptors in two independent replications.

#### 2.3.2. Data Analysis

Statistical analyses were conducted using Statistica (v.13.1, StatSoft, Tulsa, OK, USA). The effects of vegetable type (L = 8; seven vegetable types and a control sample with no vegetable), iodine form (L = 3, two iodine forms and non-iodized), and diet were analysed. The results of sensory tests were subjected to an analysis of variance (ANOVA), and then post hoc Tukey’s test was applied at a significance level of *p* < 0.05 to compare the means. Principal component analysis (PCA) was applied to the data sets from the sensory profiling of products to assess differences and similarities in sensory profiles based on their aroma, colour, and taste descriptors. PCA was also applied to the colour, aroma, taste, and overall desirability data. To determine the strength of the correlation between the variables, Pearson’s linear correlation coefficients (r) were calculated, with the results delineated as follows: r < 0.200, no linear relationship; 0.200 ≤ r < 0.400, weak linear dependence; 0.400 ≤ r < 0.700, moderate linear dependence; 0.700 ≤ r < 0.900, significant linear dependence; and r ≥ 0.900, very strong linear dependence. The significance level was set at *p* ≤ 0.05.

## 3. Results

### 3.1. Sensory Profiling Results

**Figure 1:**
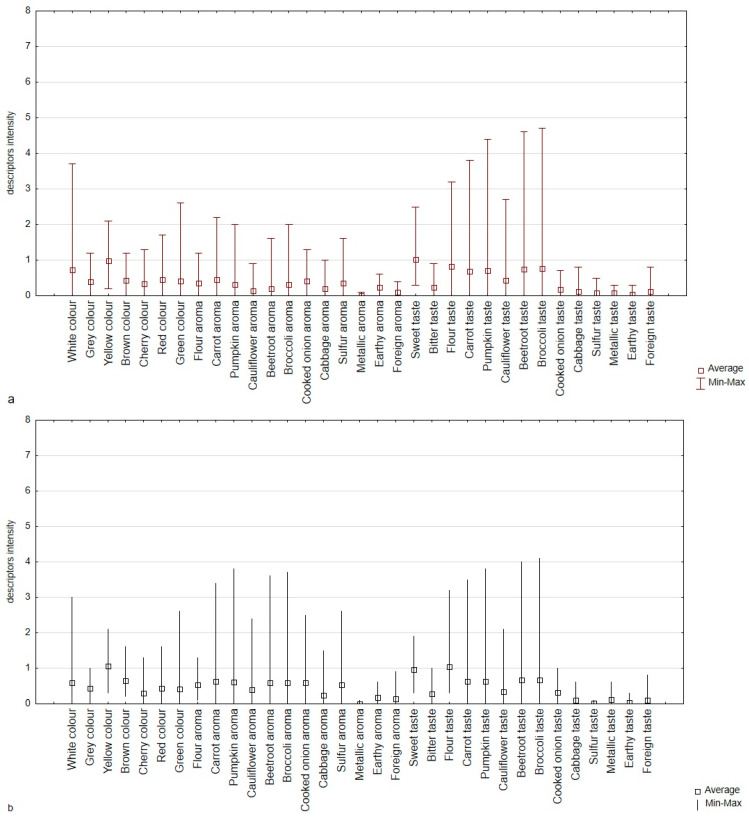
Box plot diagram of sensory profiles (colour, aroma, taste) of gnocchi dumplings (**a**) and ciabatta rolls (**b**) with the addition of iodine-fortified dried vegetables (carrot, pumpkin, cauliflower, beetroot, broccoli) and the control sample (without vegetable and iodine).

The sensory profiling defined and determined the perception of colour, aroma, and taste descriptors (Figure 1a,b, Table S1). The sensory profiling confirmed 7 descriptors for colour (white, grey, yellow, brown, cherry, red, green), 8 for aroma (flour, typical of added vegetable, cooked onion, cabbage, sulphur, metallic, earthy, and foreign), and 10 for taste (sweet, bitter, flour, typical of added vegetable, cooked onion, cabbage, sulphur, metallic, earthy, and foreign).

**Figure 2:**
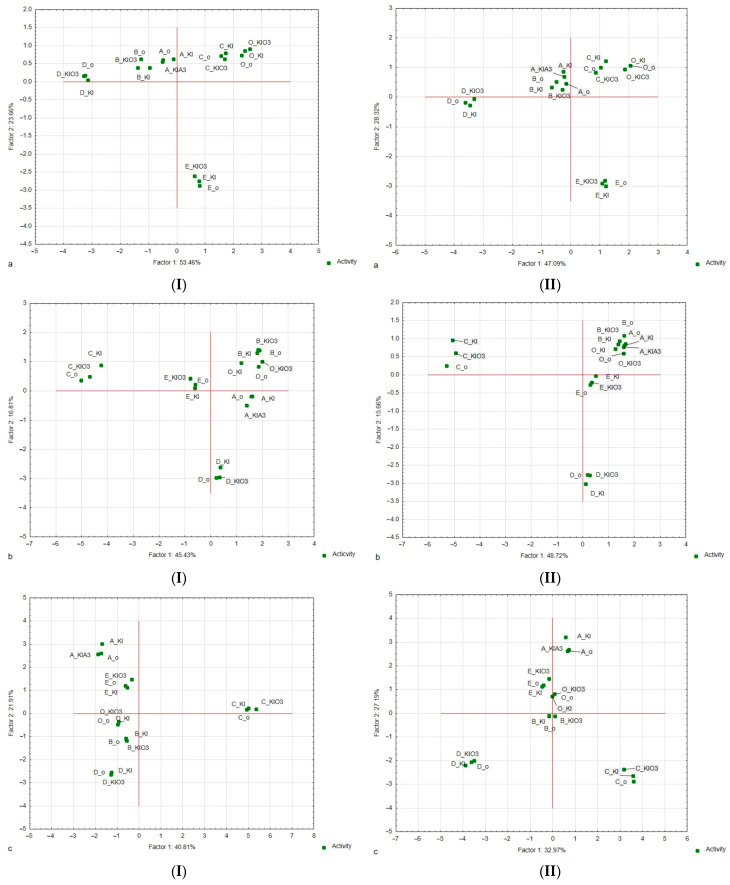
Map of the variants of gnocchi dumplings (**I**) and ciabatta rolls (**II**) with the addition of iodine-fortified dried vegetables (carrot (A), pumpkin (B), cauliflower (C), beetroot (D), broccoli (E)) and the control sample (without vegetable (O) and iodine (o)) into factors (F1 × F2). Case–factor coordinate plots based on the attributes of (**a**) colour profiles, (**b**) aroma profiles, and (**c**) taste profiles (PCA analysis).

A principal component analysis (PCA) was used to study the relations between the colour, aroma, and/or taste attributes characteristic of the sensory profiles of gnocchi dumplings and ciabatta rolls with iodine-fortified dried vegetables (variables) and to derive factors according to which these variables can be classified (Figure 2Ia–IIc).

A PCA was also used to map the variants tested in our experiment (i.e., samples with selected vegetable types and iodine form) into these factors. The PCA showed that the first two factors (F1 and F2) were the most important elements explaining variation in the data. For gnocchi dumplings, they explained approximately 77% of the total variance for colour, 62% for aroma, and 44% for taste. Those factors (F1 and F2) explained approximately 75% of the total variance for colour, 64% for aroma, and 60% for taste for ciabatta rolls. Therefore, they were selected for data interpretation.

The absolute values of factor coordinates of variables showed the relationship between the factors and the sensory attributes of the analysed gnocchi dumplings and ciabatta rolls (Table 1). For the colour attributes of both products, the first factor (F1) was most strongly related to grey, and the second factor (F2) was related to cherry and red colours. For the aroma attributes of gnocchi and ciabatta, F1 was the most strongly related to the cauliflower, cooked onion, cabbage, and sulphur aroma, and F2 was most strongly related to a beetroot and earthy (only for ciabatta) aroma. For the taste attributes of the analysed products, F1 was most strongly related to the taste of cauliflower, cooked onion, cabbage, and sulphur, and F2 was most strongly related to the taste of sweet, beetroot, and metallic (only for ciabatta).

Based on the statistical analysis, no confirmed relationship exists between the form of iodine used for enrichment (KIO_3_/KI) and the intensity of all definite descriptors.

Samples (within the same vegetable) with KI, KIO_3_, and without iodine (with non-iodized salt) were characterised by a similar intensity for all descriptors. Similar trends were confirmed for gnocchi dumplings and ciabatta rolls. This was confirmed for all descriptors of colour, aroma, and taste.

The analysis confirmed the relationship between the enrichment vegetable variant and the defined descriptors’ intensity.

For colour descriptors, the projection of the gnocchi and ciabatta variants on the factor plane F1 × F2 (Figure 2Ia,IIa) confirms that the control samples and samples with cauliflower were plotted to the right side of the F1 axis (i.e., they have positive coordinate values for F1). Samples with cauliflower were characterised by the highest (without control samples) white colour intensity. Moreover, the samples with cauliflower were characterised by the highest intensity of grey (1.0–1.2 points) and yellow (0.7–0.9 points). Samples with carrots, pumpkins, and beetroot were plotted to the left side (negative coordinate values for F1). The colour profiles of samples with carrots and pumpkins were characterised by the highest yellow intensity. The samples with beetroot addition were characterised by a yellow colour intensity and the highest brown and red colour intensity. The highest green colour intensity characterised the samples with broccoli.

For taste descriptors, the projection of the gnocchi variants on the factor plane F1 × F2 (Figure 2Ic,IIc) confirms that all samples except cauliflower were plotted to the left side of the F1 axis (i.e., they had negative coordinate values for F1).

The samples with cauliflower were plotted to the right side of the F1 axis (i.e., they had positive coordinate values for F1). These samples were characterised by the highest intensity of cabbage, metallic, cooked onion, and foreign taste.

The samples with beetroot were confirmed to have a different sensory profile for taste. These samples were plotted to the left side of the F2 axis (i.e., they had negative coordinate values for F2) and left of the F1 axis. The highest sweet taste characterised these samples. The higher taste intensity was confirmed for gnocchi samples. The high sweet taste intensity was also confirmed for samples with pumpkin. For these samples, it was confirmed that there was a higher intensity of pumpkin and carrot taste.

### 3.2. Sensory Consumer Results

The statistical analysis confirmed no relationship between the consumers’ diet type (vegan/omnivore) and the sensory desirability of the colour, both for gnocchi dumplings (*p* = 0.11) and ciabatta rolls (*p* = 0.06). The statistical analysis also confirmed no relationship between the consumers’ diet type (vegan/omnivore) and the sensory desirability of the aroma and taste desirability, both for gnocchi dumplings (in the appropriate order *p* = 0.59, *p* = 0.89) and ciabatta rolls (in the appropriate order *p* = 0.58, *p* = 0.52).

The statistical analysis also confirmed no relationship between the form of the introduced salt (without iodine/KI/KIO_3_) and the sensory desirability of the colour, aroma, taste, and overall desirability (Table 2) in all samples. This was confirmed for gnocchi dumplings (*p* = 0.60–*p* = 0.97) and ciabatta rolls (*p* = 0.70–*p* = 0.98). The statistical analysis of predictors of variance models for the sensory desirability of aroma for gnocchi-type dumplings and ciabatta-type rolls with the addition of iodine-fortified dried vegetables (Table 2) confirmed the relationship between the type of vegetable used for enrichment and the desirability of smell for both ciabatta-type rolls (F = 225.52, *p* < 0.001) and gnocchi-type dumplings (F = 271.50, *p* < 0.001).

The research confirmed that the aroma of dumplings with added dried carrot, pumpkin, broccoli, and beetroot is highly desirable (9.0–8.0 points on a 10-point scale).

It was found that the addition of dried cauliflower decreased the sensory desirability of the aroma for dumplings and rolled samples (average score of 4.5 points). This trend concerned samples with the addition of iodine-fortified and unfortified dried vegetables.

Based on the research results, the desirability of the colour was confirmed to be 8.0–9.0 points (Table S2). The statistical analysis (Table 3) confirmed that the highest correlation coefficient was between the overall desirability and the taste desirability (r = 0.99). This was confirmed for the vegan and omnivore diets. The results of the sensory analysis confirmed that the gnocchi dumplings and ciabatta rolls with iodine-fortified dried vegetables were characterised by the highest taste desirability (7.5–9.8 points) and overall desirability (8.0–9.5 points) (Table S2).

### 3.3. Relationships Between Descriptive and Consumer Desirability Data

The results of the consumer evaluation showed that gnocchi dumplings and ciabatta rolls with iodine-fortified dried vegetables were characterised by high colour, aroma, taste, and overall desirability scores (Table S2).

**Figure 3:**
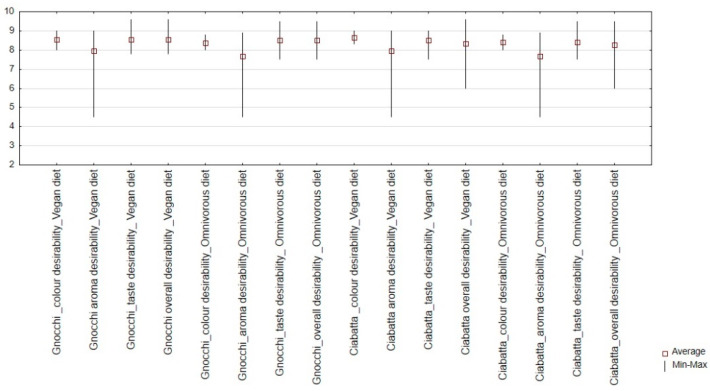
Box plot diagram of consumer desirability (colour, aroma, taste, and overall) of gnocchi dumplings and ciabatta rolls with iodine-fortified dried vegetables among consumers of vegan and omnivore diets.

The sensory analysis (Figure 3, Table S2) confirmed that vegans and omnivores have similar attitudes towards the sensory attributes of the investigated products. The statistical analysis of correlation coefficients between the intensity of analysed descriptors and consumer desirability parameters (Table 4) confirmed no relationship between the colour descriptors and the colour and overall desirability. This was confirmed for vegan and omnivore diets. The statistical analysis of the aroma desirability confirmed a negative correlation with the intensity of cauliflower aroma (vegan r = 0.980; omnivore r = 0.974), cooked onion aroma (vegan r = 0.851; omnivore r = 0.844), cabbage aroma (vegan r = 0.935; 0.916), and sulphur aroma (vegan r = 0.921; omnivore 0.901). However, the statistical analysis confirmed no relationship between these aroma descriptors (cauliflower, cooked anion, cabbage, and sulphur) and the overall desirability of the samples. The statistical analysis of the taste desirability confirmed a positive correlation with the intensity of pumpkin taste (vegan r = 0.783; omnivore r = 0.701). The statistical analysis (Table 3) confirmed that the highest correlation coefficients were between the overall desirability and the taste desirability. The analysis of correlation coefficients between the intensity of pumpkin descriptor and the overall desirability confirmed similar trends (vegan r = 0.791; omnivore r = 0.705).

## 4. Discussion

The research results confirmed that iodine addition does not influence the intensity of defined colour, aroma, and taste descriptors of gnocchi dumplings and ciabatta rolls.

Earlier instrumental studies also confirmed that the application of iodine at the quantitative level (adopted in this project) to the analysed vegetables did not affect their colour parameters (L*a*b*) [15]. Our study also confirmed the findings of earlier studies concerning the different colour profiles of products resulting from the type of vegetable. Similarly, a study comparing apple, carrot, orange pomaces, tomato, beetroot, and pumpkin flours applied in cakes had different sensory profiles [38,39]. An earlier study also confirmed the high greenness of pasta with broccoli leaves [40]. Other authors who fortified pasta products also confirmed a change in the colour parameters of pasta [41,42].

The dominance of descriptor characteristics of a given vegetable also applied to smell and taste. The results of the analysis of defined descriptors for dumplings and rolls with the addition of cauliflower confirmed the highest intensity of the smell of cooked onion, sulphur, and cabbage, and, thus, exhibited the lowest sensory desirability among both groups. Engel’s 2002 results also found this [43].

The higher intensity of the onion, cabbage, and sulphur aroma and taste in ciabatta rolls is associated with reducing the volatility of the compounds responsible for this smell. Cooking the dumplings causes these components to be washed out into water, which increases their volatility [44]. The obtained research results confirmed the highest intensity of the perceived sweet taste for products with the addition of dried beetroot.

An earlier study confirmed the relationship between a bitter taste and decreased consumer acceptance of food products, particularly those for which this taste is not characteristic [45,46]. Additionally, these earlier studies confirmed a negative correlation between a metallic taste and consumer acceptance. An earlier study showed that the problem with incorporating broccoli into food is that it can alter its sensory properties, especially its bitter taste [47,48]. Our products with vegetables were characterised by a low intensity of bitter and metallic taste, which influenced the high consumer desirability of the products.

The results of our research confirmed that gnocchi dumplings and ciabatta rolls with iodine-fortified dried vegetables were characterised by a high desirability of colour, aroma, and taste. A similar sensory desirability was also confirmed for samples without the addition of dried vegetables, both with and without the addition of iodine. Earlier studies also confirmed the sensory acceptance among vegans of dumplings with added dried vegetables [38,39]. They were also found to have high overall desirability. Hobbs’s study [40] confirmed a high overall liking for bread enriched with vegetables and bread with no vegetable inclusion. Earlier research on pasta with broccoli and pumpkin showed similar and high desirability [34,41]. Earlier research also confirmed the high desirability of cereal flour in bakery products like cakes, cookies, bread, soups, sauces, and instant noodles with the addition of pumpkin.

Our analysis confirmed the positive correlation between the intensity of the pumpkin taste descriptor and the overall desirability. An earlier study confirmed that pumpkin flour is the most popular due to its highly desirable flavour, sweetness, and deep yellow-orange colour [42]. These results confirmed only the significantly reduced desirability of bread with beetroot. Our research did not confirm a high intensity of the bitter taste descriptor for the products with beetroot. The designed products, especially dumplings, were characterised by a sweet taste. The obtained relationship may confirm the preferences of the surveyed consumers for the presence of a sweet taste in this type of product.

This manuscript provides valuable information and practical tips on explaining and possibly anticipating consumer behaviour for those following vegan and omnivore diets towards flour products with iodine-fortified dried vegetables. A strength of this study was defining the positive and negative attributes of flour products with iodine-fortified dried vegetables, with the highest impact on sensory desirability among consumers on both an omnivore and a vegan diet. Another strength was the sample size of sensory consumers, with 296 participants included. The main limitation was that the assessed products contained only potato or wheat flour as the main non-vegetable ingredient. Therefore, further research should focus on increasing the variability of the flours used (degree of milling, origin) and vegetable varieties.

## 5. Conclusions

Iodine-fortified vegetables (carrot, pumpkin, cauliflower, beetroot, broccoli) allow for the creation of new flour products with high consumer desirability. The introduction of dried iodine-fortified vegetables, at the level 24% in gnocchi dumplings and 5% in ciabatta rolls resulted in the high sensory desirability of these products. The suggests the possibility of their frequent consumption by vegan and omnivorous consumers.

The iodine content at levels of 30 μg in 100 g of gnocchi dumplings and 9–11 μg in 100 g of ciabatta rolls did not change their sensory profile. However, the high intensity of cauliflower, cooked onion, cabbage, and sulphur aroma descriptors may limit the use of these vegetables. Consumers of vegan and omnivorous diets are characterised by experiencing similar desirability for the sensory characteristics of flour products with vegetables. Taste was the sensory attribute that determines a product’s level of sensory desirability in both groups. These consumers confirmed the most positive attitude to the taste of pumpkin.

## Figures and Tables

**Table 1:** The factor loadings for colour, aroma, and taste attributes of gnocchi dumplings and ciabatta rolls with iodine-fortified dried vegetables (carrot, pumpkin, cauliflower, beetroot, broccoli) and the control sample (without vegetable and iodine).

Sensory Attributes	Gnocchi Dumplings		Ciabatta Rolls	
	F1	F2	F1	F2
Colour				
White	0.122	−0.442	0.108	0.317
Grey	0.700 *	−0.229	0.737 *	0.029
Yellow	−0.256	−0.151	−0.118	0.292
Brown	−0.541	0.756	−0.269	−0.917 *
Cherry	−0.558	0.811 *	−0.521	−0.806 *
Red	−0.572	0.798 *	−0.533	−0.793 *
Green	0.074	−0.327	−0.143	0.341
Aroma				
Flour	−0.370	−0.621	−0.312	0.539
Carrot	−0.374	−0.259	−0.222	0.348
Pumpkin	−0.272	−0.020	−0.170	0.085
Cauliflower	0.942 *	0.232	0.970 *	−0.219
Beetroot	−0.348	0.808 *	−0.405	−0.888 *
Broccoli	0.074	−0.327	−0.144	0.345
Cooked onion	0.828 *	0.369	0.912 *	−0.363
Cabbage	0.975 *	0.138	0.861 *	−0.103
Sulphur	0.975 *	0.145	0.949 *	−0.135
Metallic	−0.222	0.539	−0.224	−0.488
Earthy	0.368	0.651	0.396	−0.867 *
Taste				
Sweet	−0.453	0.860 *	−0.407	−0.770
Bitter	−0.216	−0.428	−0.269	0.517
Flour	−0.081	−0.617	−0.097	0.471
Carrot	−0.319	−0.257	−0.221	0.348
Pumpkin	−0.272	−0.020	−0.169	0.086
Cauliflower	0.944 *	0.233	0.970	−0.218
Beetroot	−0.370	0.851 *	−0.406	−0.888 *
Broccoli	0.075	−0.327	−0.144	0.344
Cooked onion	0.776 *	0.181	0.793 *	−0.020
Cabbage	0.943 *	0.235	0.967 *	−0.219
Sulphur	0.936 *	0.229	−0.226	−0.495
Metallic	0.830 *	−0.046	−0.445	−0.769 *
Earthy	−0.273	−0.250	−0.193	0.324
Foreign	0.937 *	0.235	0.945 *	−0.219

* Believed to be most important; two factors (F1 and F2) were extracted by applying PCA on the mean values of descriptive sensory scores.

**Table 2:** Statistical significance of predictors of covariance models for changes of the colour, aroma, taste, and overall desirability for gnocchi dumplings and ciabatta rolls with iodine-fortified dried vegetables among consumers following vegan and omnivore diets (one-way ANOVA test).

	SS *	*df*	MSE	F	*p*
GNOCCHI					
Colour desirability					
Vegetable type	1.24	5	0.25	3.73	0.03
Iodine Form	0.10	2	0.05	0.52	0.60
Diet type	0.25	1	0.25	2.66	0.11
Aroma desirability					
Vegetable type	40.72	5	8.15	271.50	0.00
Iodine Form	0.13	2	0.06	0.03	0.97
Diet type	0.67	1	0.67	0.29	0.59
Taste desirability					
Vegetable type	3.74	5	0.75	6.47	0.00
Iodine Form	0.06	2	0.03	0.09	0.92
Diet type	0.01	1	0.01	0.02	0.89
Overall desirability					
Vegetable type	3.74	5	0.75	6.47	0.00
Iodine Form	0.06	2	0.03	0.09	0.92
Diet type	0.01	1	0.01	0.02	0.89
CIABATTA					
Colour desirability					
Vegetable type	0.58	5	0.12	2.26	0.11
Iodine Form	0.04	2	0.02	0.24	0.79
Diet type	0.56	1	0.56	7.86	0.06
Aroma desirability					
Vegetable type	41.34	5	8.27	225.52	0.00
Iodine Form	0.18	2	0.09	0.04	0.96
Diet type	0.72	1	0.72	0.31	0.58
Taste desirability					
Vegetable type	3.02	5	0.61	17.00	0.00
Iodine Form	0.17	2	0.08	0.36	0.70
Diet type	0.10	1	0.10	0.43	0.52
Overall desirability					
Vegetable type	21.72	5	4.35	107.13	0.00
Iodine Form	0.05	2	0.03	0.02	0.98
Diet type	0.02	1	0.02	0.02	0.89

* SS—statistical significance; *df*—degrees of freedom; MSE—the standard error of the mean; F—F test value; *p*—*p*-value.

**Table 3:** Correlation coefficients between the overall desirability and colour, aroma, and taste desirability for gnocchi dumplings and ciabatta rolls with iodine-fortified dried vegetables among consumers following vegan and omnivore diets.

Sensory Atribiutes	Consumer Desirability	
	Vegan	Omnivore
Colour	0.276	0.279
Aroma	0.360	0.257
Taste	1.000	1.000

**Table 4:** Correlation coefficients between the intensity of descriptors and consumer analysis parameters of gnocchi dumplings and ciabatta rolls with iodine-fortified dried vegetables among consumers of vegan and omnivore diets.

	Consumer Desirability			
Descriptors	Vegan Diet		Omnivore Diet	
Colour	colour desirability	overall desirability	colour desirability	overall desirability
White	−0.496	−0.262	−0.405	−0.308
Grey	−0.471	−0.355	−0.323	−0.299
Yellow	−0.015	0.581	0.383	0.487
Brown	0.502	−0.050	0.113	−0.215
Cherry	0.538	0.054	0.120	−0.181
Red	0.548	0.087	0.145	−0.146
Green	0.066	0.042	0.100	0.286
Aroma	aroma desirability	overall desirability	aroma desirability	overall desirability
Flour	−0.450	0.269	−0.067	0.134
Carrot	0.297	0.247	0.253	0.202
Pumpkin	0.253	0.792	0.276	0.679
Cauliflower	−0.980 *	−0.315	−0.974 *	−0.201
Beetroot	0.257	−0.456	0.177	−0.580
Broccoli	0.194	0.042	0.242	0.283
Cooked onion	−0.851 *	−0.253	−0.844 *	−0.201
Cabbage	−0.935 *	−0.318	−0.916 *	−0.118
Sulphur	−0.921 *	−0.303	−0.901 *	−0.128
Metallic	0.137	0.114	0.192	0.109
Earthy	−0.433	−0.604	−0.497	−0.592
Taste	taste desirability	overall desirability	taste desirability	overall desirability
Sweet	−0.053	−0.062	−0.258	−0.317
Bitter	0.003	0.013	0.167	0.167
Flour	−0.081	−0.088	−0.176	−0.156
Carrot	0.051	0.079	0.063	0.066
Pumpkin	0.783 *	0.791 *	0.701 *	0.705 *
Cauliflower	−0.311	−0.321	−0.271	−0.208
Beetroot	−0.304	−0.314	−0.442	−0.491
Broccoli	0.044	0.040	0.307	0.300
Cooked onion	0.027	0.046	0.045	0.103
Cabbage	−0.322	−0.333	−0.286	−0.222
Sulphur	−0.288	−0.298	−0.237	−0.181
Metallic	−0.216	−0.227	0.052	0.101
Earthy	−0.093	−0.041	−0.020	−0.016
Foreign	−0.333	−0.343	−0.303	−0.236

* Believed to be most important.

## Data Availability

The data presented in this study are available on request from the corresponding author due to privacy reasons.

## Supplementary Materials

The following supporting information can be downloaded at: https://www.mdpi.com/article/10.3390/nu16244392/s1, Table S1. Mean scores (n = 14) of sensory colour, aroma, and taste profiling of gnocchi dumplings with the addition of iodine-fortified dried vegetables (carrot, pumpkin, cauliflower, beetroot, broccoli) and the control sample (without vegetable and iodine); Table S2. Mean scores (n = 14) of sensory colour, aroma, and taste profiling of ciabatta rolls with the addition of iodine-fortified dried vegetables (carrot, pumpkin, cauliflower, beetroot, broccoli) and the control sample (without vegetable and iodine); Table S3. Mean scores (n = 296) of sensory consumer desirability of colour, aroma, and taste of ciabatta rolls with the addition of iodine-fortified dried vegetables (carrot, pumpkin, cauliflower, beetroot, broccoli) and the control sample (without vegetable and iodine).
