# Supplementary material for: Mapping Consumer Preference for Vegan and Omnivorous Diets for the Sensory Attributes of Flour Products with Iodine-Fortified Plant-Based Ingredients
Source: Nutrients. 2024 Dec 20;16(24):4392. doi: 10.3390/nu16244392 (PMC11677174; doi:10.3390/nu16244392)
Supplement: Supplementary file 1 [file nutrients-16-04392-s001.zip › nutrients-3363681-supplementary.pdf]

Table S.1. Mean scores (n=14) of sensory colour, aroma, and taste profiling of gnocchi dumplings with the addition of iodine-fortified dried vegetables (carrot, pumpkin, cauliflower, beetroot, broccoli) and the control sample (without vegetable and iodine).

	**Variants of products**																	
Sample	O_KI	O_KIO3	O_o	A_KI	A_KIA3	A_o	B_KI	B_KIO3	B_o	C_KI	C_KIO3	C_o	D_KI	D_KIO3	D_o	E_KI	E_KIO3	E_o
Colour																		
White	3.50	3.70	3.40	0.00	0.00	0.00	0.00	0.00	0.00	0.70	0.80	0.90	0.00	0.00	0.00	0.00	0.00	0.00
Grey	0.80	0.90	0.70	0.50	0.40	0.40	0.10	0.00	0.20	1.20	1.00	1.00	0.00	0.00	0.00	0.00	0.00	0.00
Yellow	0.50	0.30	0.50	1.90	1.80	2.00	1.90	2.00	2.10	0.70	0.80	0.90	0.30	0.50	0.40	0.30	0.40	0.20
Brown	0.00	0.00	0.00	0.50	0.60	0.70	0.30	0.50	0.60	0.20	0.10	0.20	1.20	1.10	1.00	0.20	0.30	0.20
Cherry	0.00	0.00	0.00	0.20	0.30	0.20	0.60	0.50	0.50	0.00	0.00	0.00	1.10	1.20	1.30	0.00	0.00	0.00
Red	0.00	0.00	0.00	0.30	0.40	0.30	0.70	0.90	0.80	0.00	0.00	0.00	1.50	1.60	1.70	0.00	0.00	0.00
Green	0.00	0.00	0.00	0.00	0.00	0.00	0.00	0.00	0.00	0.00	0.00	0.00	0.00	0.00	0.00	2.50	2.40	2.60
Aroma																		
Flour	1.00	1.20	1.00	0.50	0.40	0.50	0.60	0.60	0.60	0.00	0.00	0.00	0.00	0.00	0.00	0.00	0.00	0.10
Carrot	0.00	0.00	0.00	2.00	2.10	2.20	0.50	0.80	0.70	0.00	0.00	0.00	0.00	0.00	0.00	0.00	0.00	0.00
Pumpkin	0.00	0.00	0.00	0.00	0.00	0.00	1.80	2.00	2.00	0.00	0.00	0.00	0.00	0.00	0.00	0.00	0.00	0.00
Cauliflow	0.00	0.00	0.00	0.00	0.00	0.00	0.00	0.00	0.00	0.90	0.80	0.90	0.00	0.00	0.00	0.00	0.00	0.00
Beetroot	0.00	0.00	0.00	0.00	0.00	0.00	0.00	0.00	0.00	0.00	0.00	0.00	1.50	1.60	0.50	0.00	0.00	0.00
Broccoli	0.00	0.00	0.00	0.00	0.00	0.00	0.00	0.00	0.00	0.00	0.00	0.00	0.00	0.00	0.00	1.90	1.80	2.00
cooked	0.80	0.00	0.00	0.00	0.00	0.00	0.30	0.40	0.30	1.20	1.30	1.20	0.40	0.30	0.50	0.20	0.30	0.20
cabbage	0.00	0.00	0.00	0.00	0.00	0.00	0.00	0.00	0.00	0.90	0.80	1.00	0.00	0.00	0.00	0.20	0.30	0.30
Sulfur	0.00	0.00	0.00	0.00	0.00	0.00	0.00	0.00	0.00	1.50	1.60	1.50	0.00	0.00	0.10	0.60	0.40	0.50
Metallic	0.00	0.00	0.00	0.00	0.00	0.00	0.00	0.00	0.00	0.00	0.00	0.00	0.00	0.00	0.00	0.00	0.00	0.00
Earthy	0.00	0.00	0.00	0.30	0.40	0.30	0.00	0.00	0.00	0.30	0.50	0.60	0.40	0.50	0.40	0.20	0.10	0.20
Foreign	0.00	0.00	0.00	0.00	0.00	0.00	0.00	0.00	0.00	0.30	0.40	0.40	0.00	0.00	0.00	0.20	0.30	0.20
Taste																		
Sweet	0.50	0.70	0.70	0.70	0.80	0.80	1.30	1.20	1.20	0.70	0.80	0.60	2.50	2.30	2.40	0.40	0.30	0.50

Bitter	0.00	0.00	0.00	0.80	0.90	0.90	0.00	0.00	0.00	0.00	0.00	0.00	0.00	0.00	0.00	0.50	0.60	0.60
Flour	2.90	3.00	3.20	0.50	0.60	0.60	0.50	0.50	0.60	0.30	0.40	0.40	0.00	0.00	0.00	0.40	0.30	0.50
Carrot	0.00	0.00	0.00	3.80	3.60	3.70	0.40	0.60	0.30	0.00	0.00	0.00	0.00	0.00	0.00	0.00	0.00	0.00
Pumpkin	0.00	0.00	0.00	0.00	0.00	0.00	3.90	4.40	4.40	0.00	0.00	0.00	0.00	0.00	0.00	0.00	0.00	0.00
Cauliflow	0.00	0.00	0.00	0.00	0.00	0.00	0.00	0.00	0.00	2.60	2.70	2.50	0.00	0.00	0.00	0.00	0.00	0.00
Beetroot	0.00	0.00	0.00	0.00	0.00	0.00	0.00	0.00	0.00	0.00	0.00	0.00	4.40	4.50	4.60	0.00	0.00	0.00
Broccoli	0.00	0.00	0.00	0.00	0.00	0.00	0.00	0.00	0.00	0.00	0.00	0.00	0.00	0.00	0.00	4.50	4.70	4.60
cooked	0.00	0.00	0.00	0.20	0.00	0.10	0.30	0.30	0.30	0.60	0.70	0.60	0.00	0.00	0.00	0.00	0.00	0.00
cabbage	0.00	0.00	0.00	0.00	0.00	0.00	0.00	0.00	0.00	0.70	0.80	0.80	0.00	0.00	0.00	0.00	0.00	0.00
Sulfur	0.00	0.00	0.00	0.00	0.00	0.00	0.00	0.00	0.00	0.50	0.40	0.40	0.00	0.00	0.00	0.00	0.00	0.00
Metallic	0.00	0.00	0.00	0.00	0.00	0.00	0.00	0.00	0.00	0.30	0.30	0.20	0.00	0.00	0.00	0.20	0.30	0.20
Earthy	0.00	0.00	0.00	0.30	0.20	0.20	0.00	0.00	0.00	0.00	0.00	0.00	0.00	0.00	0.00	0.00	0.00	0.00
Foreign	0.00	0.00	0.00	0.00	0.00	0.00	0.00	0.00	0.00	0.60	0.80	0.80	0.00	0.00	0.00	0.00	0.00	0.00

Table S.2. Mean scores (n=14) of sensory colour, aroma, and taste profiling of ciabatta rolls with the addition of iodine-fortified dried vegetables (carrot, pumpkin, cauliflower, beetroot, broccoli) and the control sample (without vegetable and iodine).

Sample	**Variants of products**																	
	O_KI	O_KIO3	O_o	A_KI	A_KIA3	A_o	B_KI	B_KIO3	B_o	C_KI	C_KIO3	C_o	D_KI	D_KIO3	D_o	E_KI	E_KIO3	E_o
Colour																		
White	3.00	3.00	3.00	0.00	0.00	0.00	0.00	0.00	0.00	0.50	0.50	0.50	0.00	0.00	0.00	0.00	0.00	0.00
Grey	0.70	0.60	0.70	0.60	0.50	0.40	0.30	0.30	0.40	1.00	0.90	0.80	0.20	0.30	0.20	0.00	0.00	0.00
Yellow	0.60	0.70	0.60	2.00	2.10	2.00	2.10	2.00	2.10	0.90	0.80	0.80	0.40	0.50	0.60	0.30	0.40	0.30
Brown	0.20	0.30	0.20	0.70	0.60	0.60	0.60	0.50	0.60	0.50	0.60	0.70	1.60	1.50	1.60	0.30	0.20	0.20
Cherry	0.00	0.00	0.00	0.40	0.40	0.30	0.20	0.30	0.20	0.00	0.00	0.00	1.10	1.20	1.30	0.00	0.00	0.00
Red	0.00	0.00	0.00	0.30	0.30	0.30	0.90	0.50	0.80	0.00	0.00	0.00	1.60	1.50	1.50	0.00	0.00	0.00
Green	0.00	0.00	0.00	0.00	0.00	0.00	0.00	0.00	0.00	0.00	0.00	0.00	0.00	0.00	0.00	2.50	2.40	2.60
Aroma																		
Flour	1.30	1.20	1.20	0.60	0.60	0.70	0.50	0.70	0.90	0.20	0.10	0.10	0.30	0.30	0.30	0.20	0.20	0.10
Carrot	0.00	0.00	0.00	3.40	3.20	3.30	0.50	0.40	0.40	0.00	0.00	0.00	0.00	0.00	0.00	0.00	0.00	0.00
Pumpkin	0.00	0.00	0.00	0.00	0.00	0.00	3.80	3.60	3.70	0.00	0.00	0.00	0.00	0.00	0.00	0.00	0.00	0.00
Cauliflo	0.00	0.00	0.00	0.00	0.00	0.00	0.00	0.00	0.00	2.40	2.30	2.40	0.00	0.00	0.00	0.00	0.00	0.00
Beetroot	0.00	0.00	0.00	0.00	0.00	0.00	0.00	0.00	0.00	0.00	0.00	0.00	3.60	3.50	3.50	0.00	0.00	0.00
Broccoli	0.00	0.00	0.00	0.00	0.00	0.00	0.00	0.00	0.00	0.00	0.00	0.00	0.00	0.00	0.00	3.50	3.70	3.50
Cooked	0.80	0.00	0.00	0.00	0.00	0.00	0.30	0.40	0.30	2.50	2.30	2.20	0.50	0.40	0.60	0.20	0.10	0.10
Cabbage	0.00	0.00	0.00	0.00	0.00	0.00	0.00	0.00	0.00	1.50	1.40	0.40	0.00	0.00	0.00	0.30	0.30	0.30
Sulfur	0.00	0.00	0.00	0.00	0.00	0.00	0.00	0.00	0.00	2.40	2.50	2.60	0.00	0.00	0.00	0.50	0.70	0.70
Metallic	0.00	0.00	0.00	0.00	0.00	0.00	0.00	0.00	0.00	0.00	0.00	0.10	0.00	0.00	0.00	0.00	0.00	0.00
Earthy	0.00	0.00	0.00	0.00	0.00	0.00	0.00	0.00	0.00	0.40	0.50	0.60	0.60	0.50	0.50	0.00	0.10	0.10
Foreign	0.00	0.00	0.00	0.00	0.00	0.00	0.00	0.00	0.00	0.90	0.80	0.80	0.00	0.00	0.00	0.00	0.00	0.00
Taste																		
Sweet	0.60	0.40	0.60	0.80	0.80	0.90	1.50	1.40	1.50	0.60	0.70	0.80	1.90	1.80	1.90	0.40	0.30	0.50

Bitter	0.00	0.00	0.00	1.00	0.90	1.00	0.00	0.00	0.00	0.00	0.00	0.00	0.00	0.00	0.00	0.60	0.70	0.60
Flour	3.00	3.20	3.00	0.80	0.90	0.90	0.90	1.00	1.00	0.40	0.60	0.50	0.40	0.30	0.40	0.50	0.60	0.40
Carrot	0.00	0.00	0.00	3.40	3.50	3.10	0.40	0.50	0.40	0.00	0.00	0.00	0.00	0.00	0.00	0.00	0.00	0.00
Pumpkin	0.00	0.00	0.00	0.00	0.00	0.00	3.60	3.80	3.80	0.00	0.00	0.00	0.00	0.00	0.00	0.00	0.00	0.00
Cauliflo	0.00	0.00	0.00	0.00	0.00	0.00	0.00	0.00	0.00	2.00	2.10	1.90	0.00	0.00	0.00	0.00	0.00	0.00
Beetroot	0.00	0.00	0.00	0.00	0.00	0.00	0.00	0.00	0.00	0.00	0.00	0.00	4.00	3.90	4.00	0.00	0.00	0.00
Broccoli	0.00	0.00	0.00	0.00	0.00	0.00	0.00	0.00	0.00	0.00	0.00	0.00	0.00	0.00	0.00	3.90	4.10	4.00
Cooked	0.00	0.00	0.00	0.30	0.50	0.50	0.40	0.60	0.40	1.00	0.90	0.90	0.00	0.00	0.00	0.00	0.00	0.00
Cabbage	0.00	0.00	0.00	0.00	0.00	0.00	0.00	0.00	0.00	0.60	0.50	0.60	0.00	0.00	0.00	0.00	0.00	0.00
Sulfur	0.00	0.00	0.00	0.00	0.00	0.00	0.00	0.00	0.00	0.00	0.00	0.00	0.10	0.10	0.10	0.00	0.00	0.00
Metallic	0.00	0.00	0.00	0.00	0.00	0.00	0.00	0.00	0.00	0.00	0.00	0.00	0.60	0.50	0.40	0.20	0.10	0.20
Earthy	0.00	0.00	0.00	0.30	0.20	0.20	0.00	0.00	0.00	0.00	0.00	0.00	0.00	0.00	0.00	0.00	0.00	0.00
Foreign	0.00	0.00	0.00	0.00	0.00	0.00	0.00	0.00	0.00	0.60	0.50	0.80	0.00	0.00	0.00	0.00	0.00	0.00

Table S.3. Mean scores (n=296) of sensory consumer desirability of colour, aroma, and taste of ciabatta rolls with the addition of iodine-fortified dried vegetables (carrot, pumpkin, cauliflower, beetroot, broccoli) and the control sample (without vegetable and iodine).

Sample	**Variants of products**																	
	O_KI	O_KIO3	O_o	A_KI	A_KIA3	A_o	B_KI	B_KIO3	B_o	C_KI	C_KIO3	C_o	D_KI	D_KIO3	D_o	E_KI	E_KIO3	E_o
Gnocchi dumplings																		
omnivore diet																		
Colour	8.00	8.50	8.00	8.50	8.50	8.50	8.50	8.70	8.50	8.00	8.50	8.40	8.00	8.50	8.50	8.80	8.00	8.50
Aroma	8.00	8.00	8.00	8.00	8.00	8.50	8.00	8.70	8.90	4.50	4.50	4.70	8.00	8.50	8.50	8.80	8.00	8.50
Taste	8.00	8.50	8.00	8.50	8.50	8.50	9.50	9.50	9.40	8.70	8.00	8.00	7.50	7.50	8.50	8.80	9.50	8.50
Overal	8.00	8.50	8.00	8.50	8.50	8.50	9.50	9.50	9.40	8.70	8.00	8.00	7.50	7.50	8.50	8.80	9.50	8.50
vegan diet																		
Colour	8.00	8.50	8.00	8.00	8.00	8.80	8.80	8.80	8.80	8.30	8.60	8.80	9.00	8.80	8.90	8.80	8.50	8.50
Aroma	8.00	8.20	8.00	8.90	8.30	8.80	8.80	8.80	8.80	4.50	4.80	4.60	9.00	8.80	8.90	8.80	8.50	8.50
Taste	8.30	8.40	8.30	8.30	8.30	9.00	9.60	9.40	9.50	8.50	8.00	8.00	7.80	7.80	8.90	8.80	8.50	8.50
Overal	8.30	8.40	8.30	8.30	8.30	9.00	9.60	9.40	9.50	8.50	8.00	8.00	7.80	7.80	8.90	8.80	8.50	8.50
Ciabatta rolls																		
omnivore diet																		
Colour	8.00	8.50	8.00	8.50	8.50	8.50	8.70	8.70	8.50	8.00	8.50	8.40	8.00	8.50	8.50	8.80	8.00	8.50
Aroma	8.00	8.00	8.00	8.00	8.00	8.50	8.00	8.70	8.90	4.50	4.50	4.70	8.00	8.50	8.50	8.80	8.00	8.50
Taste	8.00	8.50	8.00	8.50	8.50	9.00	9.00	9.00	9.50	7.70	8.00	7.50	8.00	8.50	8.50	8.50	8.00	8.50
Overal	8.00	8.50	8.00	8.50	8.50	8.50	9.50	9.50	9.40	6.20	6.00	6.50	8.00	8.50	8.50	8.80	9.50	8.50
vegan diet																		
Colour	8.30	8.60	8.30	8.30	8.30	9.00	9.00	8.80	8.80	8.30	8.60	8.80	9.00	8.80	8.90	8.80	8.50	8.50
Aroma	8.00	8.00	8.00	8.90	8.30	9.00	9.00	8.80	8.80	4.50	4.80	4.60	9.00	8.80	8.90	8.80	8.50	8.50
Taste	8.30	8.60	8.30	8.50	8.30	9.00	9.00	8.80	9.00	7.50	7.80	7.80	9.00	8.80	8.90	8.50	8.50	8.50
Overal	8.30	8.60	8.30	8.30	8.30	9.00	9.60	9.40	9.50	6.00	6.00	6.00	9.00	8.80	8.90	8.80	8.50	8.50
